# Chemical Characteristics of Three Kinds of Japanese Soy Sauce Based on Electronic Senses and GC-MS Analyses

**DOI:** 10.3389/fmicb.2020.579808

**Published:** 2021-01-06

**Authors:** Guozhong Zhao, Yixu Feng, Hadiatullah Hadiatullah, Fuping Zheng, Yunping Yao

**Affiliations:** ^1^Beijing Advanced Innovation Center for Food Nutrition and Human Health, Beijing Technology and Business University, Beijing, China; ^2^State Key Laboratory of Food Nutrition and Safety, Key Laboratory of Food Nutrition and Safety, Ministry of Education, College of Food Science and Engineering, Tianjin University of Science & Technology, Tianjin, China

**Keywords:** soy sauce, flavor, taste, GC-MS, aroma

## Abstract

Japanese soy sauce has become more acceptable by Chinese consumers due to its umami taste. However, the volatile flavor compounds and taste characters have not been fully clarified. This study aimed to explore the flavor characteristics of three kinds of Japanese soy sauce, including Koikuchi Shoyu, Usukuchi Shoyu, and Amakuchi Shoyu. The secret of volatile flavor substances was investigated by Gas Chromatography-Mass Spectrometry (GC-MS) and electronic nose, while taste compounds were investigated by silylation GC-MS and electronic tongue (E-tongue). A total of 173 volatile flavor substances and 160 taste compounds were identified. In addition, 28 aroma compounds with odor activity values (OAV) ≥ 1 were considered as the typical flavors. We found that alcohols and aldehydes were in high abundance in Japanese soy sauce, but only a small portion of pyrazines and esters were. Based on electronic nose and GC-MS analysis, Koikuchi Shoyu gives more contribution to aroma compounds, while Usukuchi Shoyu and Amakuchi Shoyu give the sourness and sweetness features based on E-tongue and silylation GC-MS analysis. In this study, 50 kinds of sugars were detected that contributed to the sweetness of soy sauce. This study will provide new insight into the flavor characteristics of Japanese soy sauce that potentially contribute to the innovation and development of soy sauce.

## Introduction

Soy sauce, produced mainly in China or Japan, is widely consumed as a food condiment or seasoning in Asian countries and other neighboring countries, to improve the appetite, desirable flavor, and digestion. The quality of soy sauce may determine its acceptability to consumers, which has given manufacturers a new challenge. Nowadays, Japanese soy sauce has attracted more attention due to its unique and high-quality flavor. The fermentation method of soy sauce has been modified in Japan, but it is still traditional in China today. Japanese soy sauce has improved the brewing process, which has been introduced in some factories in China.

Soy sauce is commonly made from soybeans and wheat, fermented by *Aspergillus oryzae* for koji, with the addition of 18% saline water for moromi. Lactobacilli and yeast are selectively used during moromi fermentation (Kataoka, 2005). Three typical varieties of Japanese soy sauce favored in different areas of Japan, such as Koikuchi Shoyu (eastern Japan), Usukuchi Shoyu (midwestern Japan), and Amakuchi Shoyu (southern Japan), are well recognized by Chinese consumers. Furthermore, these kinds of soy sauce can be easily purchased in China from offline or online markets, such as Taobao. Koikuchi Shoyu is dark-colored with a slightly fruity flavor and is made from a high percentage of soybeans, which reduces fishy and meaty smells in cooking. It is suitable for stew meat and barbecue seasoning. Usukuchi Shoyu has a lighter color and saltier taste than Koikuchi Shoyu. It is commonly used for udon noodle soup, chawanmushi, and simmered seasoning (nimono). Amakuchi Shoyu is a light-colored and sweet soy sauce that represents an ideal match with seafood seasoning. However, the differences in flavor compounds lead to a complicated usage scale of these kinds of soy sauce.

Several important aroma compounds of soy sauce have been detected. 5(or 2)-ethyl-4-hydroxy-2(or 5)-methyl-3(2*H*)-furanone (4-HEMF), a caramel-like aroma, is one of the most essential aroma compounds in soy sauce, and it was also detected in Koikuchi Shoyu and Usukuchi Shoyu (Kaneko et al., 2012). Volatile phenols such as 4-vinylguaiacol (4-VG) and 4-ethylguaiacol (4-EG) are produced by *Candida versatilis* during moromi fermentation (Suezawa and Suzuki, 2007). In our previous study, some typical fragrant compounds with aromatic rings, such as phenylacetaldehyde, 2-phenylethanol, and phenylethyl acetate, accounted for a large proportion of the flavors of traditional Chinese-type soy sauce (Zhao et al., 2020). To date, over 400 flavor compounds have been detected in soy sauce, and other more important flavors are continually being discovered (Meng et al., 2014). The flavor and taste compounds of the three kinds of Japanese soy sauce were not fully clarified in the previous study. Therefore, this investigation of the flavor and taste is necessary and will help us to improve the flavor of our products. The research will contribute to the innovation of traditional industries in China.

In this study, an electronic nose and electronic tongue (E-tongue), combined with GC-MS, were used to determine the secret of volatile flavor substances and taste compounds of Koikuchi Shoyu, Usukuchi Shoyu, and Amakuchi Shoyu, which could uncover the mystery of Japanese soy sauce for different kinds of dishes.

## Materials and Methods

### Materials

Three kinds of Japanese soy sauce were purchased online (Taobao) and divided into three groups. Koikuchi Shoyu was named S1, Usukuchi Shoyu S2, and Amakuchi Shoyu S3. GC-grade of 2-octanol and N, O-bis(trimethylsilyl)trifluoroacetamide and trimethylchlorosilane (BSTFA + 1% TMCS, 99:1) were purchased from Sigma (Sigma Aldrich Co., St. Louis, MO, United States). N, N-dimethylformamide (DMF) (99%) was purchased from damas-beta (Adamas Reagent, Ltd.). Heptadecanoic acid (98%) was purchased from Alfa Aesar (Ward-Hill, MA, United States).

### Electronic Nose Analysis

Portable electronic nose 3 (PEN 3, Airsense Co., Germany), which assembles 10 sensors for different application targets, was applied for detection. A soy sauce sample (5 ml) was carefully transferred into the sealed bottle for electronic nose (E-nose) detection and then soaked in a water bath (50°C) for 20 min. The cleaning process was maintained for 220 s to exclude impurities. After being cleaned to the baseline, the samples were detected and persisted for 120 s until sensors reached stable values. The headspace flavor compounds were pumped into the sensor chamber at a constant rate of 300 ml/min. The cleaning procedure was repeated after completion of the detection. Each sample was carried out in triplicate.

### GC-MS Analysis

A soy sauce sample (5 ml) was carefully transferred into a bottle with sufficient headspace (20 ml) with a small magnetic stir bar. Compound 2-octanol was selected and injected into the bottle as the internal standard substance. Then, the bottle was soaked in a water bath (60°C) with a magnetic stirring apparatus (250 rpm) (IKA, RCT Basic, Staufen, Germany). After 30 min balancing, the cap seal was punctured by a 50/30 μm DVB/CAR/PDMS extraction fiber (Supelco, Inc., Bellefonte, PA, United States), which was exposed above the soy sauce sample for absorption for 30 min. Then, the solid-phase microextraction (SPME) fiber was removed and inserted into the heated injector of GC-MS (QP2010 Ultra, Shimadzu Co., Kyoto, Japan) with 15 min desorption time. The separation was performed with a low-polarity Rtx-5ms column (30 m × 0.25 mm × 0.25 μm) (Restek Co., Bellefonte, PA, United States). The column temperature was programed at 40°C for 3 min and then increased to 150°C at a rate of 4°C/min for 1 min. At the final stage, the temperature was increased to 250°C at a rate of 8°C/min for 6 min with the nitrogen as the carrier (N2 99.99%, 3.5 ml/min). The ionization voltage of the mass spectrometer (MS) was set at 70 eV. The test voltage was 0.8 kV, and the scan area was 29–500 u. Flavor compounds were identified based on RI by comparing the experimental spectra of reference standards with matches in the standard NIST 17 library. The similarity of flavor compounds was detected over 80% of the standard spectra library.

### Electronic Tongue Analysis

Electronic tongue Taste-Sensing system SA 402B (Intelligent Sensor Technology Co. Ltd., Japan) equipped with five test sensors (CA0, C00, AE1, CT0, and AAE) and two reference electrodes Cai was used in this study following Cai et al. (2020). The taste sensors were, namely, CA0 specific for sourness, C00 for bitterness and aftertaste bitterness (aftertaste-b), AE1 for astringency and aftertaste astringency (aftertaste-a), CT0 for saltiness, and AAE for umami and richness (aftertaste of umami). The sensor array was initially immersed into a sample (30 ml) as the reference solution, then continuously treated into other sample solutions for 120 s. The sensor array was rinsed with a sensor cleaning solution containing 5% ethanol until a stable potential was obtained. Each sample was carried out in triplicate.

### Silylation Derivatization Procedure and GC-MS Detection

A soy sauce sample (50 μl) was transferred into a 1.5 ml EP tube and frozen for 6 h at -80°C, and then treated by vacuum freeze-drying for 12 h. DMF (100 μl) (Sigma, United States) was added to dissolve the freeze-dried sample. The mixture was treated by an ultrasonic oscillator (YH-200DH, Shanghai, China) for 15 min and then vibrated with a vortex oscillator (Vortex-Genie 2, United States) for 5 min to make the sample fully dissolved. Finally, the sample was centrifuged at 12,000 rpm for 3 min. The supernatant (50 μl) was transferred into a new EP tube and then mixed with 100 μl N, O-Bis(trimethylsilyl)trifluoroacetamide with 1% trimethylchlorosilane (BSTFA + 1%TMCS, 99:1) (Sigma, United States) and 5 μl heptadecanoic acid solution (the internal standard substance, dissolved in DMF solution, 10 mg/ml). After being heated in the water bath (70°C) for 2 h, 1 μl sample was absorbed after cooling down to the room temperature for GC-MS analysis (Agilent Technologies, Santa Clara, CA, United States). Helium was used as a carrier gas at a constant flow rate of 1 ml/min. Oven temperature was maintained at 70°C for 8 min, programed at 3°C/min to 190°C and then raised to 300°C with a rate of 10°C/min and held for 11 min. The MS was operated in electron impact mode with the electron energy set at 70 eV and a scan range of 43–1,000 m/z (scan rate: 4.37 scans/sec, gain factor: 1, resulting EM voltage: 1,188 V). The temperatures of MS source and quadrupole were set at 230 and 150°C, respectively. The components of the compounds were preliminarily identified according to the published data, retention time, and mass spectra of real compounds. The substances with a similarity of more than 70% were selected.

### Statistical Analysis

All tests were conducted in triplicate; significant differences were tested by analysis of variance (ANOVA) using SPSS 17.0 (SPSS Statistics, Chicago, IL, United States) at *p* < 0.05, and Tukey’s test was applied to compare average values. Data analysis of the electronic nose was based on pattern recognition software (Win Muster) provided by the PEN3 e-nose device (Schwerin, Germany). Principal component analysis (PCA) was also achieved by using SPSS 17.0. PLSR analysis was based on Unscrambler 10.4 (CAMO ASA, Oslo, Norway). The rest of the other figures were drawn by Origin 8.5 (OriginLab, MA, United States).

## Results and Discussion

### Koikuchi Shoyu Showed Significant Contribution in Aroma by E-Nose Analysis

**FIGURE 1:**
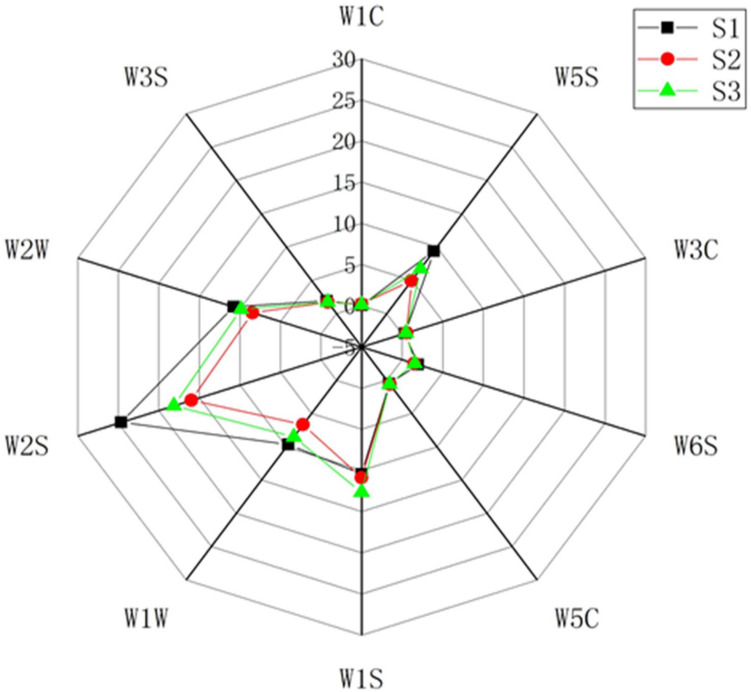
E-nose analysis of 3 kinds of Japanese soy sauce.

The total volatile compounds of soy sauce samples were distinguished by E-nose (Figure 1). PCA and loading analysis (LDA) were used to discriminate these samples according to the differences between volatile compounds and signal intensities. The migration of different samples along principal components 1 and 2 (PC1 and PC2) accounted for 96.04 and 3.36% of the total variance, respectively (Supplementary Figure 1A). The measurement results overlapped; however, the degree of separation between the samples was noticeable. These three groups of samples were clearly distinguished by LDA, which showed the significant differences among S1, S2, and S3 (Supplementary Figure 1B). The 10 important sensors were compared by LDA, which is useful for identifying the important sensors according to the values of loading parameters for a particular principal component, which might be responsible for discrimination in the current pattern file (Gao et al., 2017). W2S, W1S, W1C, W5S, W1W, and W2W had much greater influence on the current pattern file than the others, with the contribution of W2S being the most obvious sensor for characterization (Supplementary Figure 1C). The results showed that Koikuchi Shoyu had more advantages in aroma than two other Japanese soy sauce, Usukuchi Shoyu and Amakuchi Shoyu. The W2S sensor was more sensitive in S1 than others to compounds such as alcohols, aldehydes, and ketones, indicating that more oxygenated compounds exist in Koikuchi Shoyu. W5S was sensitive to nitrogen oxides, which are also indicated in Koikuchi Shoyu. The relatively high response values of W1W and W2W in S1 indicate that sulfides play a vital role in Koikuchi shoyu. Low sensitivity to methane or methyl substances was found in Koikuchi shoyu, but its benefit remains in the aroma.

### Aroma Compounds Were More Typical in Koikuchi Shoyu by GC-MS Analysis

**FIGURE 2:**
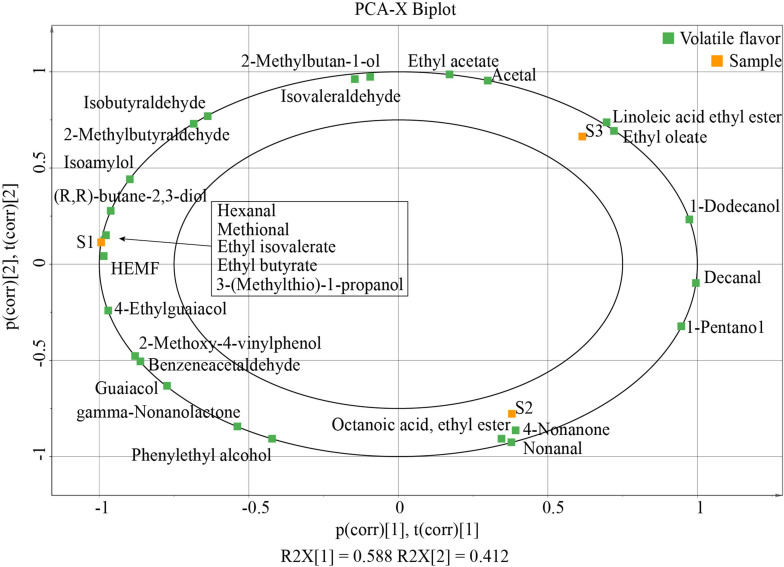
Score plot of principal components 1 and 2 for aroma compounds by PCA.

A total of 173 volatile flavor compounds were detected, including 35 alcohols, 60 esters, 26 aldehydes, 10 acids, 22 ketones, 8 phenols, 4 ethers, and 8 heterocycles (Supplementary Table 1). Significant differences in the contents of volatile compounds were found among these soy sauces. Alcohols were the most abundant volatile compounds in S1 and S3, followed by esters and aldehydes. In all, 28 aroma compounds with OAV ≥ 1 were focused and analyzed by PCA (Table 1). Figure 2 showed the two-dimensional score plot (PC1 and PC2), which accounted for 58.8 and 41.2% of the variation, respectively. Flavor compounds were clearly separated, and some typical flavor substances closely related to the three kinds of Japanese soy sauce were revealed. As shown in Figure 2, S1 had a significant correlation with HEMF, 4-EG, 2,3-butanediol, 3-methylthio-1-propanol, ethyl butyrate, ethyl isovalerate, methional, and hexanal, while S2 was correlated with octanoic acid ethyl ester, 4-non-anone, and nonanal. Only two flavors of linoleic acid ethyl ester and ethyl oleate were closely related to S3.

**TABLE 1:** Flavor compounds with OAV ≥ 1 in soy sauce.

**CAS**	**Compounds**	**Concentration (mg/l)**			**Odor threshold**	**OAV**		
		**S1**	**S2**	**S3**		**S1**	**S2**	**S3**
112-53-8	1-Dodecanol	0.04	0.14	0.20	0.016	2.28	8.84	12.64
123-51-3	Isoamylol	1.40	0.43	0.75	0.004	351.11	108.20	186.34
137-32-6	2-Methylbutan-1-ol	0.64		0.41	0.0159	40.42		25.75
24347-58-8	(R,R)-butane-2,3-diol	3.90	0.20	0.78	0.0951	41.03	2.15	8.18
505-10-2	3-(Methylthio)-1-propanol	0.22			0.12323	1.81		
60-12-8	Phenylethyl alcohol	2.07	2.19	1.66	0.56423	3.67	3.89	2.94
71-41-0	1-Pentano1		0.19	0.15	0.1502		1.29	1.03
104-61-0	gamma-Nonanolactone	0.03	0.03		0.0097	3.13	3.48	
105-54-4	Ethyl butyrate	0.00			0.0009	4.23		0.00
108-64-5	Ethyl isovalerate	0.01			0.00001	721.85		
141-78-6	Ethyl acetate	1.37	0.36	2.51	0.005	273.53	71.24	502.49
544-35-4	Linoleic acid ethyl ester	0.24	2.03	11.65	0.45	0.53	4.51	25.88
106-32-1	Octanoic acid, ethyl ester		0.03		0.0193		1.59	
111-62-6	Ethyl oleate		0.19	1.32	0.87		0.21	1.51
105-57-7	Acetal	0.04		0.11	0.0049	8.10		22.89
112-31-2	Decanal	0.01	0.04	0.04	0.003	3.36	12.82	13.25
122-78-1	Benzeneacetaldehyde	1.35	1.24	1.06	0.0063	213.62	196.75	168.71
3268-49-3	Methional	0.11			0.00045	251.92		
590-86-3	Isovaleraldehyde	1.44	0.90	1.64	0.0011	1307.73	819.42	1491.83
66-25-1	Hexanal	0.01			0.005	1.13		
78-84-2	Isobutyraldehyde	0.89		0.62	0.0015	596.35		413.57
96-17-3	2-Methylbutyraldehyde	1.57	0.55	1.92	0.001	1565.32	552.94	1922.52
124-19-6	Nonanal		0.02		0.0011		22.61	
27538-09-6	HEMF	0.11	0.08	0.07	0.00115	93.53	66.37	64.48
4485-09-0	4-Nonanone		0.31		0.0082		38.26	
2785-89-9	4-Ethylguaiacol	1.94	1.16	0.69	0.0695	27.82	16.65	9.99
7786-61-0	2-Methoxy-4-vinylphenol	6.58	4.56	1.44	0.01202	547.32	379.65	120.10
90-05-1	Guaiacol	0.07	0.06		0.00048	151.30	115.86	

4-Hydroxy-2(or 5)-ethyl-5-(or 2)-methyl-3(2H)-furanone and 2-methylbutyraldehyde give the caramel-like aroma and malty aroma, respectively, and were detected in all these samples with high OAV (Lee et al., 2006). These substances could enhance the flavor performance of soy sauce when used in cooking. Another typical aroma compound, ethyl butyrate, a short-chain ester, plays a significant role in soy sauce as aroma constituents with fruity flavors (pineapple, passion fruit, and strawberry), which can enrich the flavor of soy sauce (Rodriguez-Nogales et al., 2005; Kaneko et al., 2012). Ethyl isovalerate was a derivative of valeric acid found in fruits with a fruity odor reminiscent of blueberry (Mahmood et al., 2013). And 3-methylthio-1-propanol contributed a unique burnt, caramel-like, garlic, and cooked potato aroma to the soy sauce. Hexanal was positively associated with the cooked grain aroma (Silva et al., 2010). These aroma compounds may contribute a significant reason that S1 is favorably used for stew meat and barbecue condiment.

### The Comparison of Aroma Compounds in Japanese Soy Sauce and Chinese Traditional Soy Sauce Based on the Literature

The advantage of the flavor compounds in Koikuchi Shoyu is described in the previous study (Kaneko et al., 2012). Lots of alcohols were detected in Japanese soy sauce (Supplementary Table 1), more than in Chinese traditional soy sauce. The content of ethanol in Japanese soy sauce is much higher than Chinese traditional soy sauce (average >three times). The varieties of aldehydes in Japanese soy sauce are highly related to the artificially inoculated strains. The formation of the branched aldehydes, such as isobutyraldehyde, isovaleraldehyde, and benzeneacetaldehyde with floral and malty flavors, is probably related to the transamination of the amino acids by yeast or lactic acid bacteria (Gocke et al., 2007). Pyrazines, such as 2,6-dimethylpyrazine, trimethylpyrazine, 2-ethyl-6-methylpyrazine, 3-ethyl-2,5-dimethylpyrazine, and 2-methyl-pyrazine—the special compounds with the burnt flavor in Chinese traditional soy sauce—were relatively higher than in Japanese soy sauce (Zhao et al., 2020). These compounds may be attributed to the high temperature during moromi fermentation. Esters were also found in Chinese traditional soy sauce in abundance, such as ethyl lactate and triethyl citrate that give it a fruity flavor. In addition, some esters, such as phenethyl acetate and ethyl phenylacetate, were unique to Chinese soy sauce (Ding et al., 2019). Esters are commonly recognized as important flavor and fragrance molecules in soy sauce due to their typically fruity smell and high volatility.

### Sourness and Sweetness as the Indicators of Soy Sauce by E-Tongue Analysis

**FIGURE 3:**
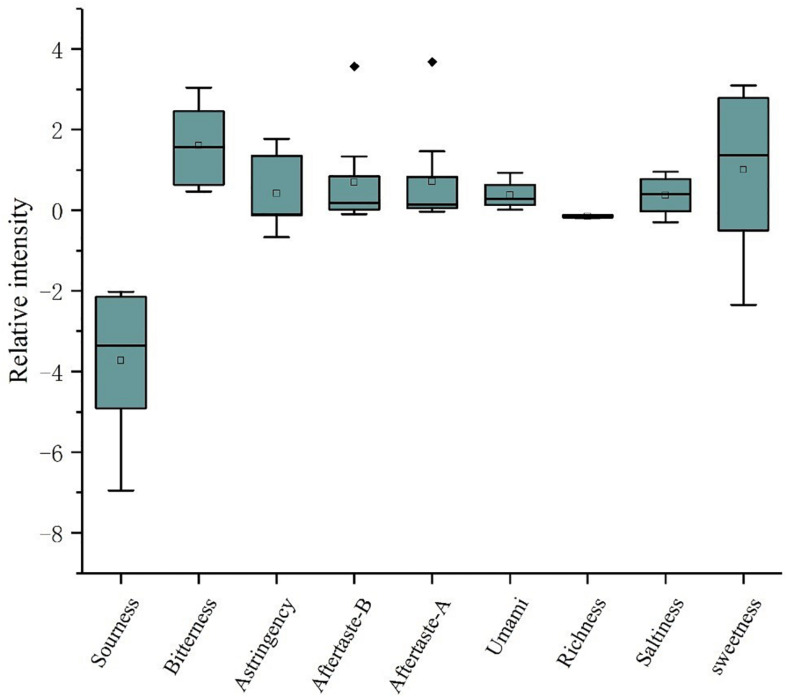
Box-plot figure of the relative intensities of taste indexes measured by E-tongue in Japanese soy sauce.

Electronic tongue quantitatively analyzed the six basic soy sauce tastes, sourness, bitterness, astringency, saltiness, umami, and sweetness, by using artificial lipid membrane sensor technology (Kobayashi et al., 2010). Sensor work time was estimated at 200 s to reach the optimum state for effective evaluation of the differences of soy sauce samples (Haddi et al., 2014). The box-plot of all variable sensors is shown in Figure 3. We can see that the soy sauce samples included in this study display great differences in the two indicators of sourness and sweetness, and extremely different values were reached at 4.93 and 5.45, respectively, which indicate that sourness and sweetness are the main factors in distinguishing these three types of soy sauce by taste attribute. The results may be closely related to taste of sugars, amino acids, fatty acids or organic acids.

### Overview of the Silylation GC-MS Analysis

In this study, a non-targeted analysis of taste components was performed by silylation GC-MS. A total of 160 non-volatile compounds were detected, including 10 amino acids, 8 amines, 36 organic acids, 50 sugars, 21 esters, 20 alcohols, and 6 ketones (Supplementary Table 2). This method was desirable for its simplicity, precision, and speed, especially for semiquantitative and qualitative analysis. The significant differences in organic acids and sugars were consistent with the results of E-tongue, indicating that fermentation technologies or fermented strains affect the metabolic carbohydrate features during soy sauce fermentation (EI Sheikha and Hu, 2020). Stearic acid and palmitic acid were found to be major components in soy sauce, which are identified as saturated fatty acids and mainly come from soybean oil. Generally, the oxides of fatty acids lead to unpleasant flavor and taste, but the complex system of soy sauce includes various substances that cover each other up or affect the flavor jointly. Therefore, the high levels of fatty acids may lead to a fuller flavor.

### Non-volatile Acid Compounds in Soy Sauce by Silylation GC-MS Analysis

A total of 33 organic acids were found in soy sauce by silylation GC-MS. Although the total organic acid content in S1 was lower than in S2 and S3, the varieties of organic acids were abundant in S1. Lactic acid, a common acidic agent and taste regulator, was the predominant organic acid in soy sauce samples. The lactic acid content was 6008.52 mg/l in S2, which was over 2.4 times more than S3 and over 6 times more than S1. Lactic acid can be produced by the fermentation of sugars (sucrose, glucose, fructose and so on) using lactic acid bacteria or yeast. Succinic acid was rich in S1 and S3, which enhanced umami taste to these two kinds of soy sauce. The 2-keto-L-gulonic acid content in S2 was 3,068 mg/l, but it was not detected in S1 and S3. The high content of 2-keto-L-gulonic acid also indicated that the different stains worked during soy sauce fermentation. S2 and S3 are usually used for seafood condiments to reduce the fishy smell because of their unique acids.

### Usukuchi Shoyu and Amakuchi Shoyu Accounted for Far More Sugars Than Koikuchi Shoyu by Silylation GC-MS Analysis

**FIGURE 4:**
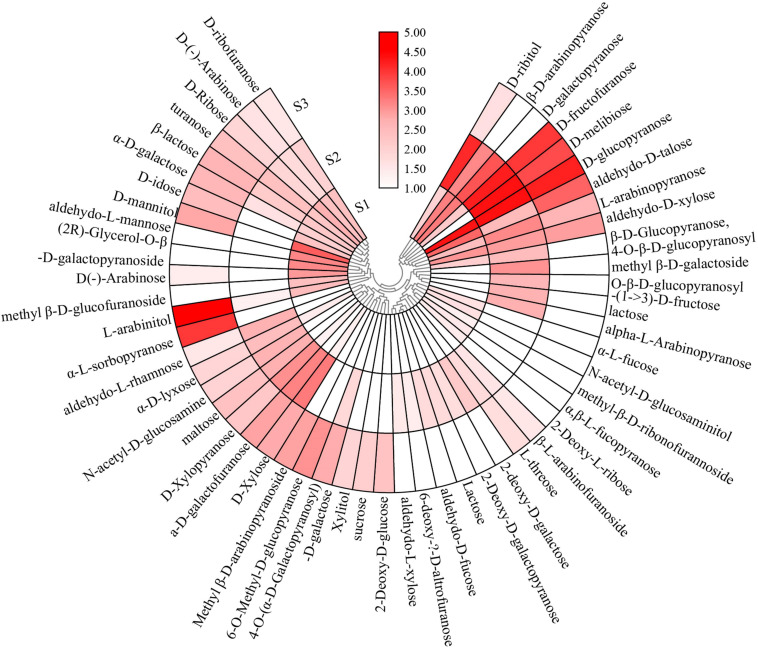
The heat map of sugars detected in Japanese soy sauce.

Sweetness is another key indicator to distinguish the three kinds of soy sauce. Fifty types of sugars were detected in this study. Sugars were typical taste compounds due to their abundant content—49.9, 52.2, and 45.3% of S1, S2, and S3, respectively. Sugars favorably contribute to the sweetness of soy sauce. The sugar content of S2 was prominent at 83,668 mg/l, and S3 accounted for 51,709 mg/l, whereas S1 was much lower. D-melibiose’s content highly contributed to the taste of S2, accounting for 23,353 mg/l, but only 6,037 mg/l in S3 and none in S1. D-fructofuranose accounted for more ratios in S2 and S3 (Figure 4). D-fructofuranose can be obtained by enzymatic transformation of glucose and hydrolysis of sucrose.

Sugars such as L-arabinopyranose and D-xylose are mainly obtained from wheat bran, the raw material of soy sauce, and showed higher content in S2 than others. Some sugars may be released from enzymolysis. D-ribofuranose was distributed through all the Japanese soy sauce samples with a lower content that released from the hydrolysis of polysaccharides (Wesener et al., 2015). Turanose, a structural isomer of sucrose, is a high-quality functional sweetener (Wang et al., 2012). In addition, rare sugars, such as D-xylopyranose, α-D-lyxose, and α-D-galactose, were also detected in this study, which plays important role in the diet, health care, medicine, and other fields. D-xylopyranose and α-D-lyxose were prominent in S2, but α-D-galactose was more abundant in S3.

### The Relationship Between Amino Acids and Aromas in Soy Sauce

Amino acids are essential taste compounds released by proteolysis of the raw materials (Zhao et al., 2016). Amino acids, such as glycine, alanine, and threonine, provide a sweet taste. In this study, glycine and alanine contents in S1 and S3 were higher than in S2, but not threonine. The possible interaction of glycine, alanine, and threonine may also lead to a strong taste of soy sauce (Heyer et al., 2003). Lysine in S2 tastes sweet/bitter. While phenylalanine, valine, leucine, and serine taste bitter (Yu et al., 2015). GABA (4-aminobutyric acid), a non-protein amino acid and bioactive component, was detected in S1 and S2 (Dhakal et al., 2012). The content of GABA in S2 was seven times higher than in S1. The results showed that L-pyroglutamic acid (L-PGA) contents were higher in S1 and S3, but not L-glutamic acid. L-PGA is a tasteless compound converted from L-glutamine by a non-enzymatic pathway (Kim and Lee, 2008), which yields a faster browning process in soy sauce (Wegener et al., 2017). In this study, the presence of L-PGA may be generated due to heat treatment during silylation.

**FIGURE 5:**
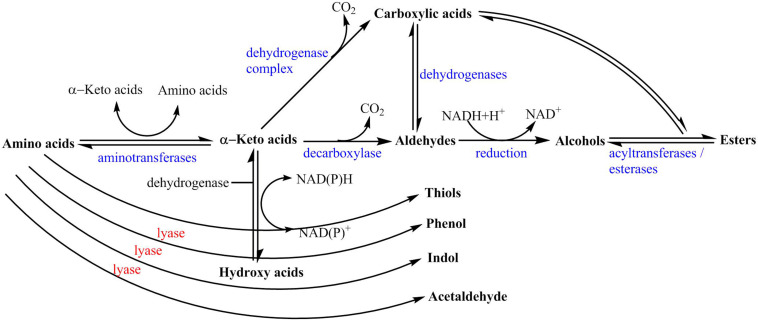
Conversion relationships between amino acids and other aroma compounds.

Amino acids are the main sources of aroma substances that are closely related to some alcohols and aldehydes. As shown in Figure 5, amino acids can be deaminated by aminotransferase to a-keto acids, which act as the precursors of higher alcohols. Also, amino acids can be converted to aldehydes by decarboxylase. For example, leucine can be converted to a-ketoisocaproic acid by amiotransferase, then converted to 3-methylbutanal by decarboxylase, and finally reduced to 3-methylbutanol by Ehrlich pathway (Smit et al., 2004). Simultaneously, valine, phenylalanine, and threonine can be degraded to 2-methylpropanal, phenylacetaldehyde, phenylethanol, 2-methylbutyraldehyde, and 2-methylbutanol. Additionally, amino acids also have close relationships with the formation of volatile compounds such as pyrazines. In this study, 2, 6-dimethylpyrazine, 2-ethyl-6-methylpyrazine, and trimethylpyrazine were obtained by the conversion of serine, threonine, and lysine. In addition, 2-phenylethanol, important volatile alcohol, can be degraded from phenylalanine by amino acid decarboxylases.

## Conclusion

Soy sauce has become a worldwide condiment for its unique flavor and taste for cooking. But some differences existed between soy sauce samples for different fermentation technologies, strains, or raw materials. Alcohols and aldehydes is highly abundant in Japanese soy sauce, which has only a modest range of pyrazines and esters. Based on the electronic nose and GC-MS analysis, Koikuchi Shoyu contributes more to flavor compounds. Usukuchi Shoyu and Amakuchi Shoyu have sourness and sweetness features according to E-tongue and silylation GC-MS analysis. Additionally, 50 kinds of sugars were detected that provide the sweetness of soy sauce. This study will provide remarkable knowledge about flavor and taste improvement of soy sauce, which potentially can improve the quality of soy sauce in the future.

## Data Availability Statement

The original contributions presented in the study are included in the article/Supplementary Materials, further inquiries can be directed to the corresponding authors.

## Author Contributions

GZ: writing, reviewing, editing, and supervision. YF: writing of the first draft and investigation. HH: methodology. FZ and YY: conceptualization and project administration. All authors contributed to the article and approved the submitted version.

## Conflict of Interest

The authors declare that the research was conducted in the absence of any commercial or financial relationships that could be construed as a potential conflict of interest.
