# Supplementary material for: Chemical Characteristics of Three Kinds of Japanese Soy Sauce Based on Electronic Senses and GC-MS Analyses
Source: Front Microbiol. 2021 Jan 6;11:579808. doi: 10.3389/fmicb.2020.579808 (PMC7815529; doi:10.3389/fmicb.2020.579808)
Supplement: Supplementary file 3 [file Table_3.DOCX]

**Figure S1.** E-nose analysis of the 3 kinds of Japanese soy sauce. A: PCA analysis; B: LDA analysis; C: Loading analysis.


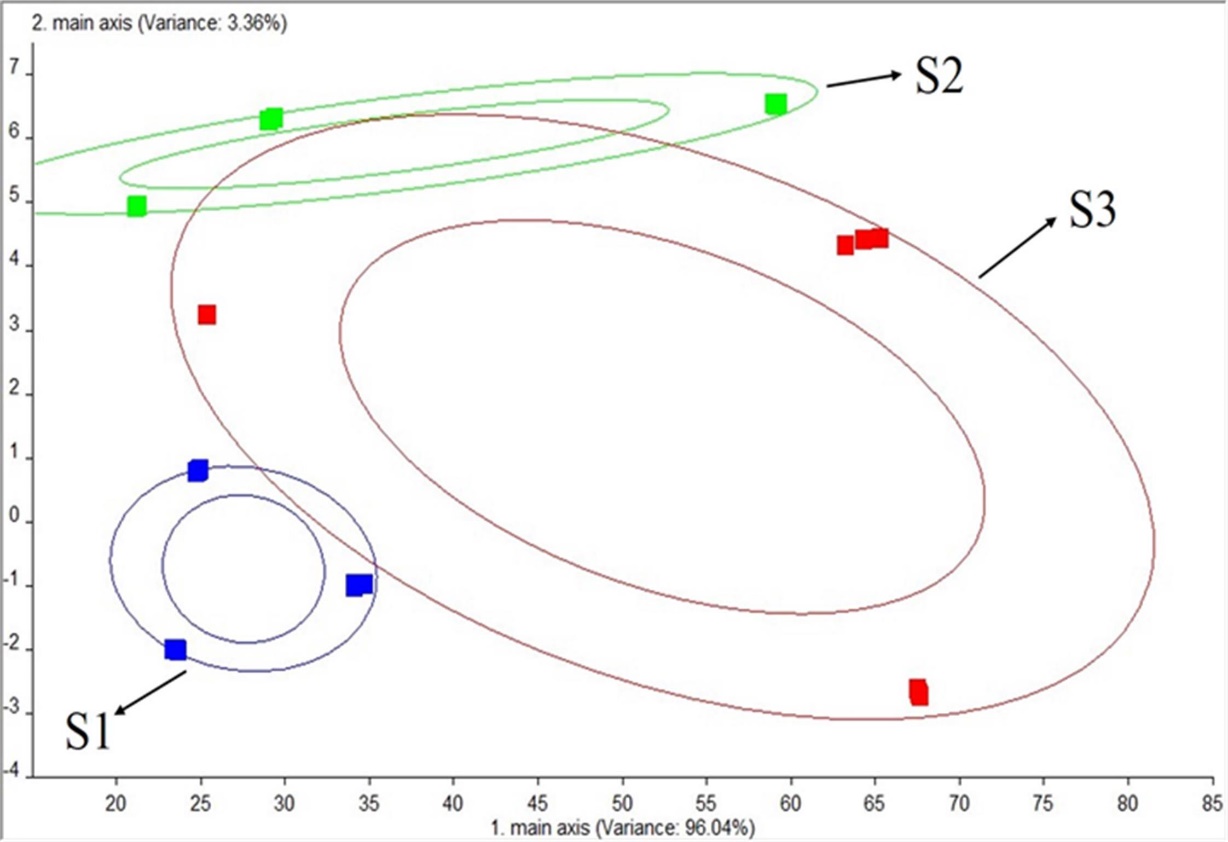


A


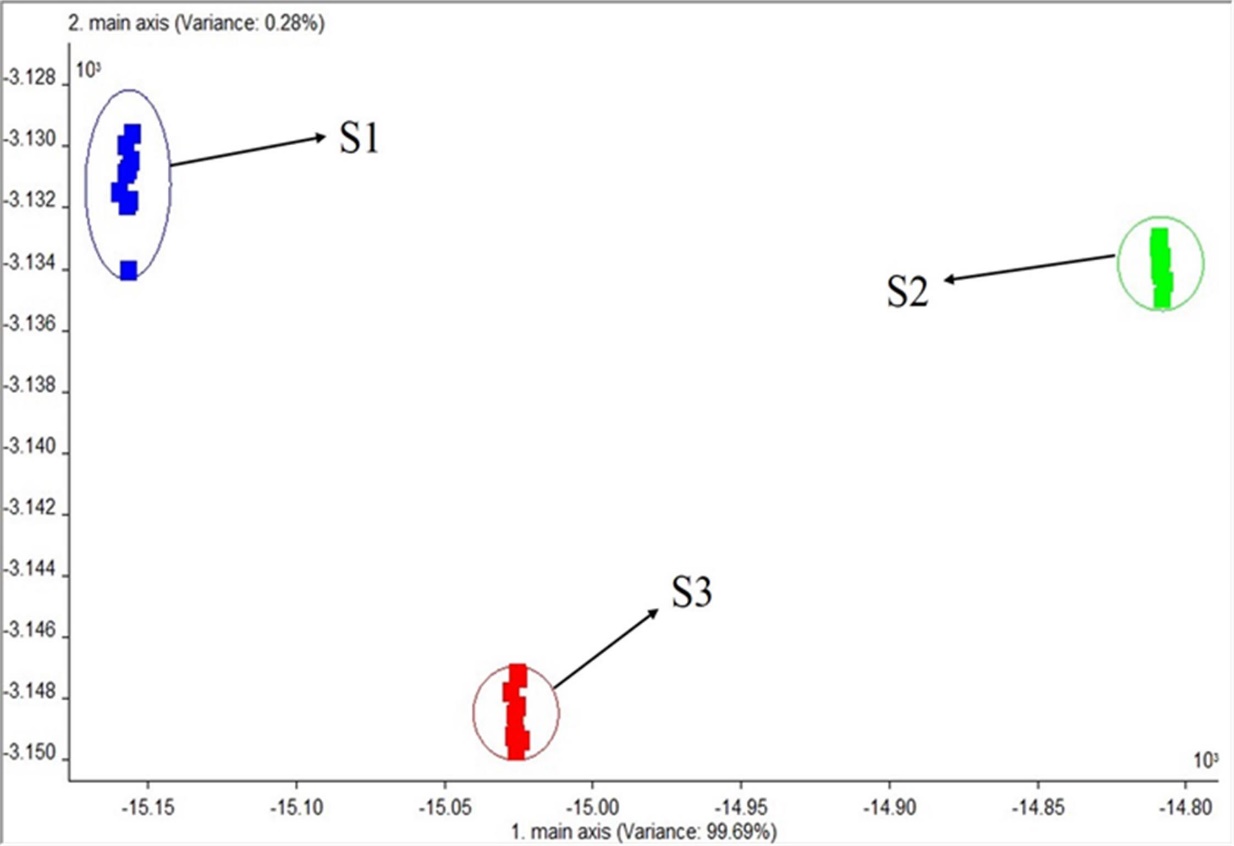


B


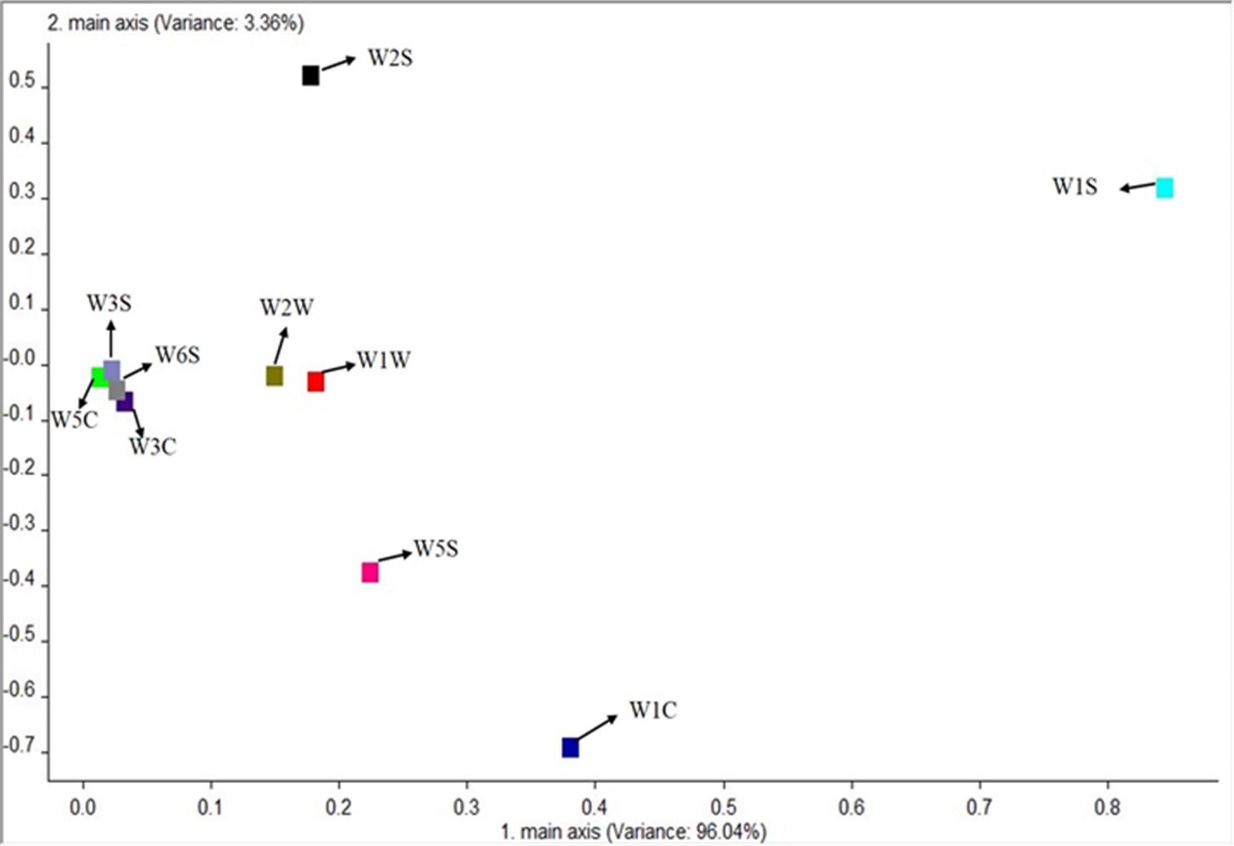


C
